# Thermal Relationship in Tropical Anurans from Two Contrasting Habitats Along an Elevation Gradient in Colombia

**DOI:** 10.21315/tlsr2024.35.1.12

**Published:** 2024-03-30

**Authors:** Katalina Gutiérrez Hernández, Carlos Alberto Galindo Martínez, Jorge Luis Turriago González, Manuel Hernando Bernal Bautista

**Affiliations:** Biology Department, Faculty of Sciences, Tolima University, Ibagué, Tolima, Colombia

**Keywords:** Altitude, Amphibians, Conservation, Microhabitats, Temperature

## Abstract

Anurans are ectothermic organisms highly susceptible to variations in the environmental temperature that changes with elevation and between habitats in tropical mountains. The aim of this study was to evaluate the variation of body temperature (BT) of nocturnal anurans from two contrasting habitats (open and forest habitats) along an elevation gradient in Colombia. We measured the environmental temperatures (substrate and air) and BT of 135 adult frogs of 11 species from open and forest habitats at three elevational zones of an Andean Mountain. The BT had a positive and significant relationship with environmental temperatures and showed a higher thermal dependence for substrate than air temperature, which suggests that anurans are thermoconformers and potentially tigmotherms. Additionally, BT of anurans from both habitats decreased with the elevation, but species from open habitats had a higher BT than forest species. Therefore, the impact of environmental temperatures on anurans that live at a similar altitude level is not the same, as the type of habitat has a strong influence on their BT. This information is important to a better understanding of anuran thermal biology, refine conservation strategies, and to improve the predictive power of environmental data in forecasting the effects of climate change on small ectotherms such as amphibians.

**Highlights**

- Substrate temperature (Ts) was notably a better predictor of tropical anuran body temperature (BT) than a close air temperature (Ta).
- Anurans from high elevation (2,700 m.a.s.l) had a lower BT than those from lowlands (455 m.a.s.l), as expected by the temperature lapse rate.
- Anurans from open habitats had a higher mean BT, although not necessarily a wider BT variation, than species from forest habitats.

## INTRODUCTION

Environmental temperatures play an important role in the physiological and behavioral functions of amphibians, as they mostly regulate their body temperatures (Angilletta 2009; Duellman & Trueb 1994; Hillman *et al*. 2008). Additionally, local variation in environmental temperatures may determine the selection of microhabitats where amphibians can carry out successfully their biological activities (Nowakowski *et al*. 2017). In tropical mountains, the thermal environment is affected by elevation due to the gradual decrease in air temperature as elevation increases, the adiabatic cooling (Ehlers & Poulsen 2009). The thermal environment can be also modified by climatic change and habitat conversion which generally increase the temperatures (Hussain & Pandit 2012; Nowakowski *et al*. 2017; Nowakowski *et al*. 2018; Root *et al*. 2003). Particularly, at a microclimate scale, variations in the cloud and vegetation cover, wind speed and relative humidity, strongly influence the convection and conduction of microenvironmental temperatures (Gates 1980), which are the two main mechanisms of heat exchange in nocturnal amphibians (Pough *et al*. 2015). Consequently, changes in environmental temperatures may alter the geographical distribution, temporal activity, physiological and behavioural performance of amphibians, predominantly in endemic and thermal specialised species as those that inhabiting the Andean mountains of Colombia (Bernal & Lynch 2013; Lynch & Suárez 2002).

The thermal ecology of amphibians has been studied extensively for many years (Brattstrom 1963; Iturra *et al*. 2014; Navas *et al*. 2013; Taylor *et al*. 2020). However, there is limited information on the body temperatures (BT) of tropical anurans in relationship to microenvironmental temperatures, which differ significantly from macroclimate data that have been demonstrated to have limited predictive power in small animals, such as amphibians (Sanabria & Quiroga 2019). Therefore, information on average temperature and thermal variation of microhabitats seems indispensable to understand amphibian BT in tropical mountain systems, beyond the known phenomenon of BT decreasing with elevation (Navas *et al*. 2013). These microclimate data are especially important in the Andes because they are a global hot spots of anuran species richness and endemism (Armesto & Señaris 2017; Ortiz *et al*. 2013), and because Andean amphibians are very susceptible to decline in response to climate change (Catenazzi *et al*. 2014; Corn 2005; Delgado & Burrowes 2022; Hussain & Pandit 2012; Iturra *et al*. 2014; Navas *et al*. 2013).

In this work, we recorded the environmental temperature and BT of tropical anurans from two contrasting habitats along an elevation gradient in an Andean Mountain of Colombia. Specifically, we evaluated the influence of: (1) air and substrate temperatures (Ta and Ts), (2) elevation, and (3) type of habitat (open vs forest) on BT of nocturnal anurans. As amphibians are considered tigmotherms (Cruz *et al*. 2016; Duellman & Trueb 1994), we expected that BT would show a stronger association with Ts than Ta, thus indicating to be a better predictor of variations in activity temperature of anurans. We also expected a negative relationship between BT and elevation, in response to environmental temperature decreasing as elevation increases (Navas *et al*. 2013; Ramírez *et al*. 2009). Finally, we hypothesised that anurans from open habitats (which are daily exposed to greater solar radiation) would have a higher mean and thermal variation than those from forest habitats along the studied elevational gradient. We hypothesised this result given the thermal stability of forest environments (Bohlman *et al*. 1995; Navas *et al*. 2013). Therefore, this paper aims to contribute to a better understanding of the thermal variation in two contrasting habitats (forest and open) across elevation and its impact on anuran BT.

## MATERIALS AND METHODS

### Study Sites and Species

**Figure 1:**
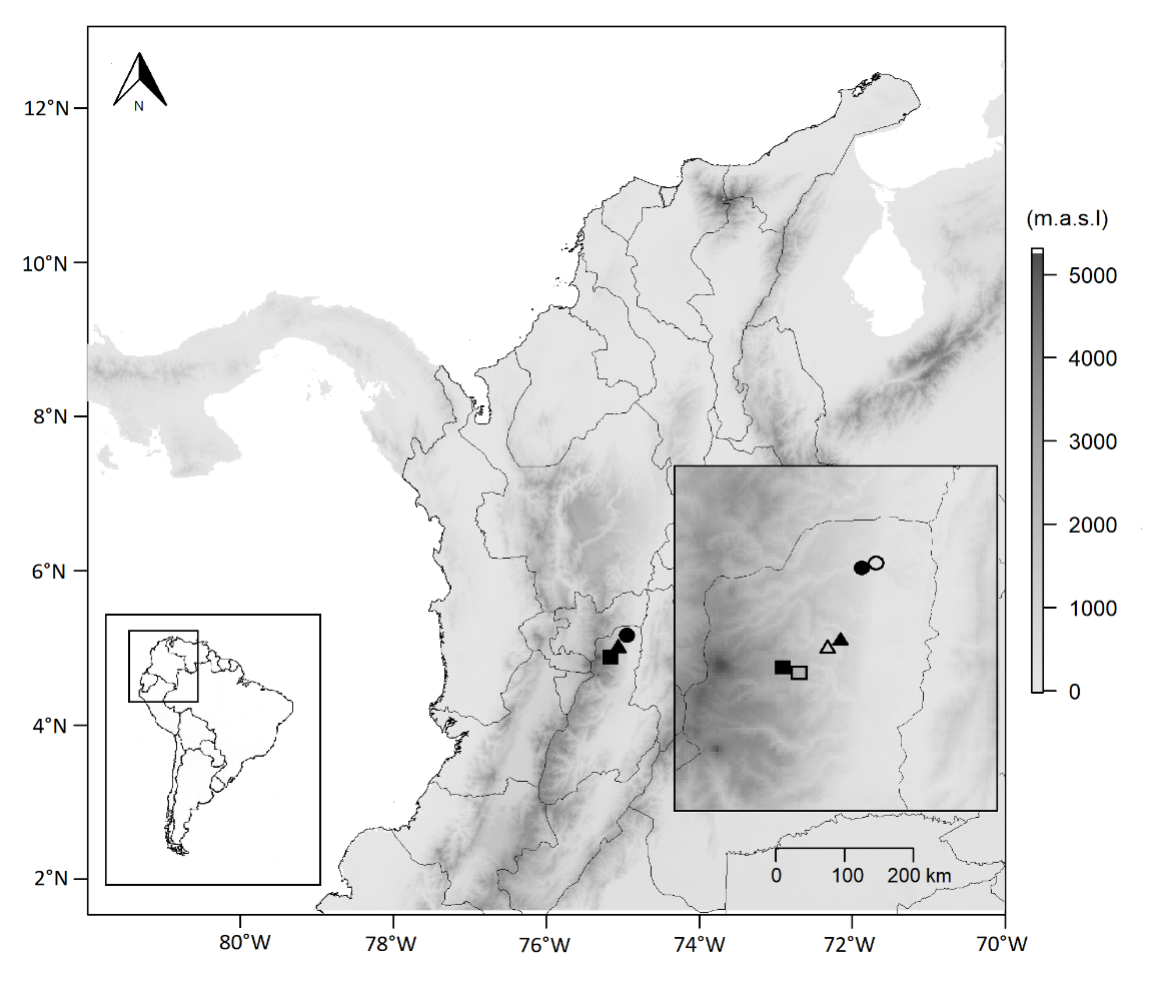
Location of study sites in the department of Tolima, Colombia. Black circle: forest area in Mariquita (5°11′25″ N, 74′54″58″ W); white circle: open area in Mariquita (5°10′18″ N, 74°53′59″ W). Black triangle: forest area in Líbano (4°57′14″ N, 75°00′05″ W); white triangle: open area in Líbano (4°55′56″ N, 75°01′39″ W). Black square: forest area in Murillo (4°52′25″ N, 75°08′54″ W); white square: open area in Murillo (4°52′28″ N, 75°09′08″ W).

This work was carried out at three different elevations (455 m.a.s.l, 1,400 m.a.s.l and 2,700 m.a.s.l) along the Cordillera Central of Colombian Andes, at the Tolima department, Colombia. The specific locations studied and their coordinates are detailed in Fig. 1. At each elevation, during three consecutive nights per month, from September to December 2018, two people actively searched adult anurans, based on acoustic and visual encounters (Angulo *et al*. 2006), both in forests as well as in open habitats, at approximately the same nocturnal hours, from 18:00 h to 24:00 h. Thus, our cumulative sampling effort was approximately of 72 h per person. We defined a forest habitat when the associated vegetation was mainly arboreal, with a close canopy higher than 60% (in which the land surface is covered by tree canopies), while a habitat was considered open when it had a maximum canopy cover (open canopy) up to 40% (FAO 2001; Galeana *et al*. 2009).

### Measures of Microenvironmental and Anuran Body Temperatures

Temperatures from anuran microhabitats were obtained with six data loggers IButton (DS1921G, accuracy of ± 1°C), which were placed in both the forest and open habitats for each of the three elevational zones studied (2 × 3 = 6 IButtons). Specifically, each data logger we positioned on the ground, in direct contact with the soil surface, where adult anurans were found. The data loggers were programmed to register temperatures each hour from September to December 2018, broadly coinciding with rainy season and frog samplings.

Anuran BT and environmental temperatures (Ts and Ta) were measured simultaneously in the field with two thermometers Extech (421502, precision 0.3°C, 0.05% accuracy) and their two thermocouples type K. The BT was taken over the dorsal part of animal, whereas Ts was recorded directly in the place where the animal was found, such as on the surface of the ground, trunks, leaf litter, soil vegetation (pastures, mosses) or leaves of trees. When some animals were found in water, Ts was recorded as the temperature of the water surface. The Ta was measured 20 cm above the animal. Previously, in the laboratory of Herpetology at the Tolima University, at different hours of the day, we simultaneously measured 10 times the skin temperature and cloacal temperature (with the thermocouple type K and thermometer Extech) of nine animals from four species with different body sizes, so as to evaluate the differences between external and internal temperatures. We did not find significant differences (Repeated Measures ANOVA, *p* > 0.05 in all cases), as it has been reported in other amphibians (Cruz *et al*. 2016; Oromí *et al*. 2010). Therefore, we assumed that skin temperature reported in this study represents the internal body temperature of anurans. We also measured the snout-vent length of anurans (SVL) using a digital caliper (Mitutoyo, precision 0.01 mm).

### Statistical Analyses

We used descriptive statistics (mean ± standard deviation, range) to show the thermal environmental variables and BT. We conducted linear regressions and Pearson correlations to evaluate separately the relationship between BT and environmental temperatures (Ta and Ts), as data met the assumption of normality (Shapiro-Wilks test, *p* > 0.05). Additionally, we performed an ANCOVA to compare the BT of anurans from open vs forest habitats at each of the three elevational zones studied, using the SVL as covariable, and the Levene’s test to compare the variances of BT between these two habitats. All statistical analyses were realised in RStudio version 4.1.2.

We recognise the importance of phylogenetic analyses for interspecific comparative studies; however, some previous data suggest that elevation and natural history may influence BT more than shared ancestry (Navas *et al*. 2013). In addition, due to the limited number of species studied, our data are likely not suitable for phylogenetic analysis (Blomberg *et al*. 2003). For that reason, herein we analysed our data employing conventional statistical analyses independent of phylogenetic position.

## RESULTS

Data on the species studied, anuran BT and environmental temperatures for the forest and open habitats along the elevational gradient sampled are shown in Table 1. Mean BT varied from 11.0°C in *Pristimantis permixtus* at 2,700 m.a.s.l to 25.2°C in *Rhinella margaritifera* at 455 m.a.s.l. The highest BT variation within species was 3.1°C (range: 23.7°C–26.8°C) for *R. margaritifera* from the open habitat at 455 m.a.s.l, whereas the lowest one was 1.5°C (range: 10.0°C–11.5°C) for *P. permixtus* from the forest habitat at 2,700 m.a.s.l (Table 1).

**Figure 2:**
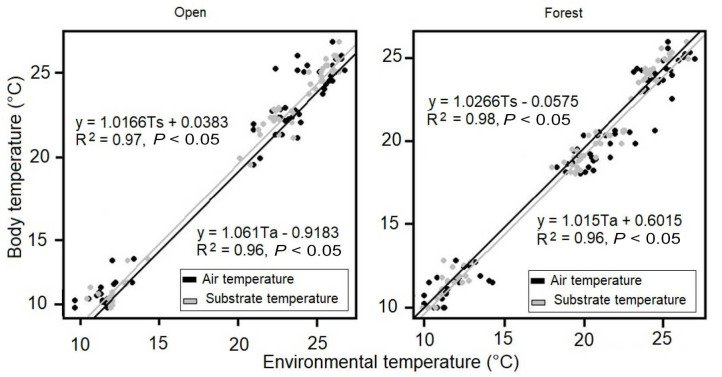
Linear regression between BT and environmental temperatures in two contrasting habitats (open and forest) along an elevational gradient in the Cordillera Central of Colombia. °C: Celsius degree.

**Figure 3:**
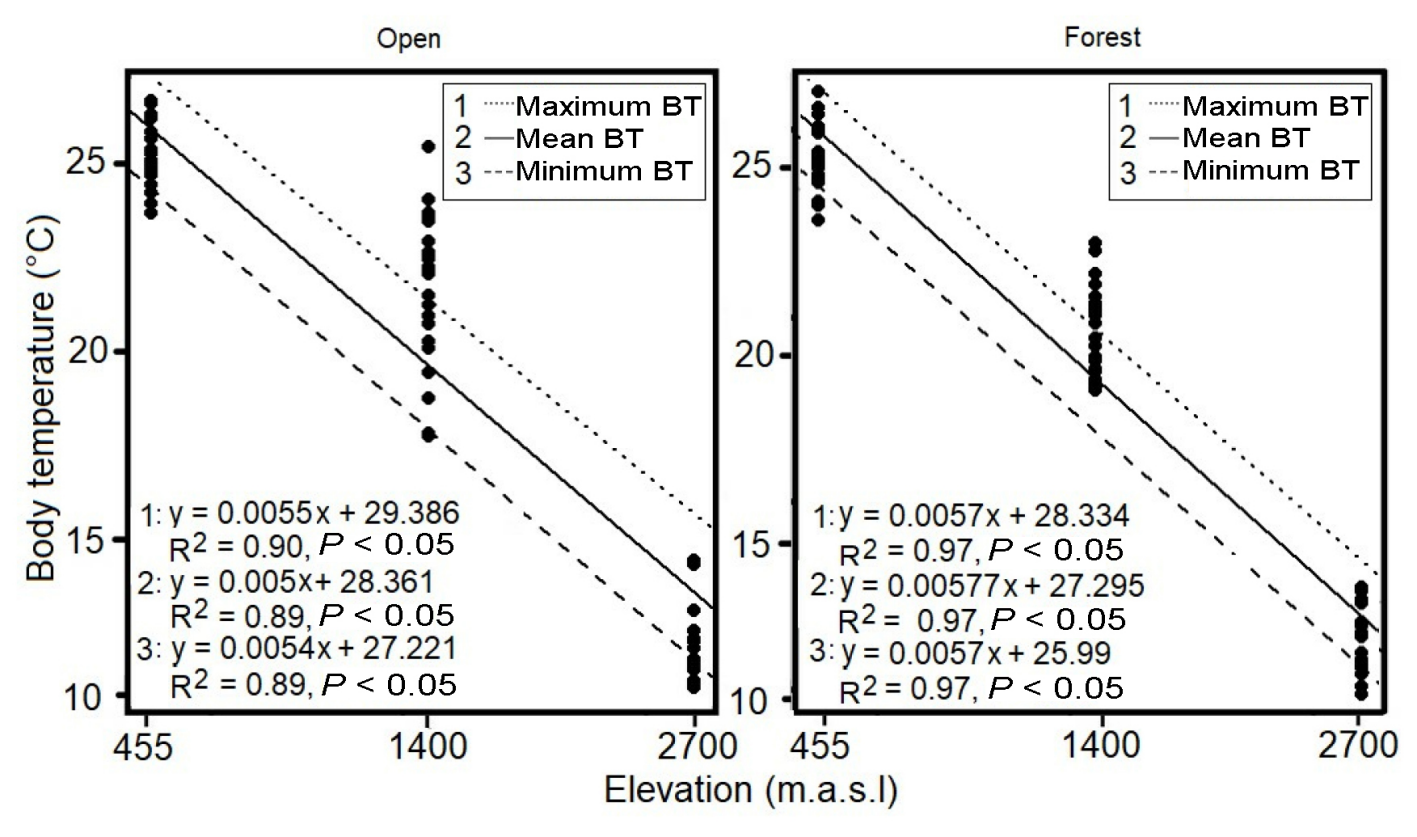
Linear regressions of anuran BT from two contrasting habitats (open and forest) as a function of elevation in the Cordillera Central of Colombia. °C: Celsius degree.

In both open and forest habitats, BT had a positive and significant relationship with environmental temperatures (Fig. 2). Particularly, Pearson’s test was higher than 0.65 for the relationship between BT and Ts in all cases, whereas it was lower than 0.65 between BT and Ta (Table 2). Body temperature of anurans (mean, maximum and minimum) decreased significantly with elevation in both open and forest habitats (Fig. 3). Similarly, Pearson correlation coefficient (r) showed a high inverse linear relationship between BT and elevation in both habitats studied (open habitat, r = −0.94; forest habitat, r = −0.99).

**Figure 4:**
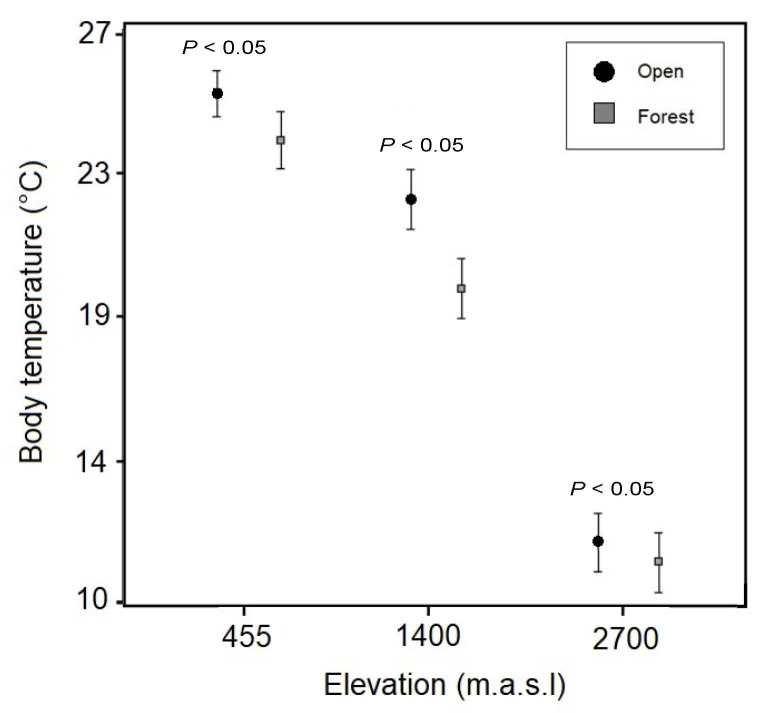
Body temperatures of anurans from two contrasting habitats (open and forest) at three elevational zones in the Cordillera Central of Colombia. The circles and squares indicate the mean and the lines the standard deviation. C°: Celsius degree.

**Figure 5:**
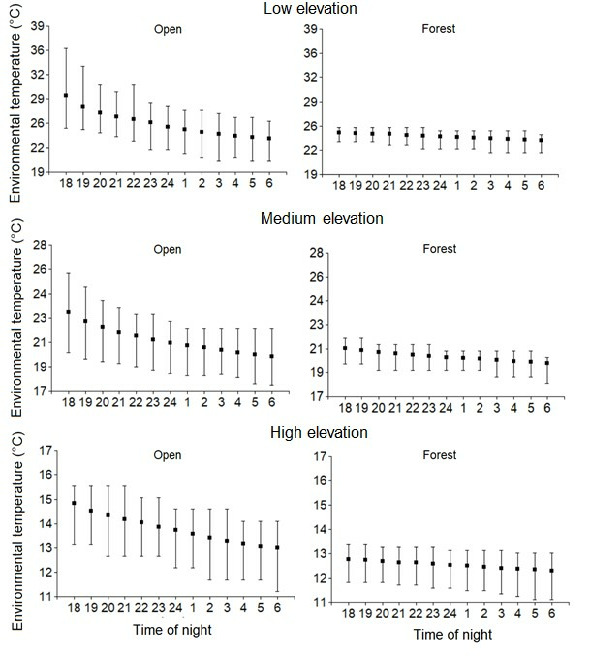
Range of maximum and minimum nocturnal temperatures (squares indicate the mean) from 18:00 h to 6:00 h in two contrasting habitats along three elevational zones. °C: Celsius degree.

Anurans from open habitat had a significantly higher BT than those from forest habitat, controlling for differences in body size (ANCOVA, F = 66.36, *p* < 0.001). However, the variance of the BT was not significant between open and forest habitats (Levene’s test, *p* > 0.05). Particularly, comparing the anuran BT between these two habitats but within each elevational zone studied, the difference was statistically significant for the species from both low and medium zones (Tukey test *p* < 0.05), but not for those from the high elevation (Tukey test *p* > 0.05) (Fig. 4). Nevertheless, microenvironmental nightly temperatures (from 18:00 h to 6:00 h) showed higher hourly mean temperatures and greater range of variation for open than forest habitats in all three elevational zones studied (Fig. 5).

## DISCUSSION

The positive and significant relationship between anuran body and environmental temperatures in two contrasting habitats in an Andean elevational gradient in Colombia (Fig. 2), indicates that frogs have the tendency to thermoconformity (i.e., BT parallels changes in environmental temperatures), as has also been reported in other related amphibian studies (Carvajalino *et al*. 2011; Iturra *et al*. 2014; Rueda *et al*. 2016). This thermal strategy results important because it may reduce the water loss maintaining the skin wet (Duellman & Trueb 1994) while amphibians perform their routine activities, such as foraging, calling, breeding and resting (Sanabria *et al*. 2011; Sanabria *et al*. 2014). Additionally, the stronger correlation of BT with Ts than Ta (Table 2), suggests that anurans present a tigmothermal regulation, where the conduction is probably the main way of heat exchange (Lara & Luja 2018; Leyte *et al*. 2018), as seen in other nocturnal amphibians, such as *Pleurodema thaul* (Iturra *et al*. 2014), and nine of the eleven species studied by Lara and Luja (2018). Thus, these data demonstrate that Ts is a better predictor of anuran BT than Ta, similar to that found in other amphibian species, *Leptodactylus ocellatus* (Sanabria *et al*. 2003), *Pleurodema thaul* (Iturra *et al*. 2014), and most of the anurans studied by Navas *et al*. (2013). This is an important finding for generating more realistic models about the effects of elevation, or environmental variables, on the physiological and behavioural performance of amphibians, especially when unusual or abrupt thermal changes may have a strong value in terms of thermal ecology and conservation of amphibians.

As expected, BT of anurans decreased through elevation in both habitats (open and forest) (Fig. 3), like other neotropical salamanders (Feder & Lynch 1982), and amphibians from the eastern Nepal Himalaya (Khatiwada *et al*. 2020), and the tropical Andes (Navas *et al*. 2013; Pintanel *et al*. 2019). This trend may be explained by the thermoconformity of amphibians which follows the inverse relationship between microenvironmental temperatures and elevation (Khatiwada *et al*. 2020; Navas *et al*. 2013). This result also suggests that elevation may have a stronger impact on BT than phylogeny (Navas *et al*. 2013), as this comparison was made among different taxa and within the same species located at different elevation, e.g., *B. platanera* and *R. margaritifera*, with similar outcomes, a lower anuran BT at the highest elevation (Table 1). Furthermore, the effect of differences in temperatures between two elevations was clearly more noticeable on anuran BT than the effect of differences in temperature between the two microhabitats studied at the same elevation, even though microhabitats can generate small areas with stable environmental conditions which may diverge from this main trend (Jones *et al*. 2011; Pintanel *et al*. 2019). For instance, the minimum BT difference between two elevational zones was 3.2°C in open habitats (Mariquita: 455 m.a.s.l and Líbano: 1,400 m.a.s.l: 25.1°C–21.9°C), whereas the maximum BT difference between forest and open habitats was 2.6°C (21.9°C–19.3°C) in Líbano (Tolima), the intermediate elevational zone (Table 1). Then, in this work we detected that changes in elevation caused a higher effect on anuran BT than microhabitat temperatures.

The mean of anuran BT was significantly higher in open than forest habitats at the same elevational level (450 and 1,400 m.a.s.l) (Fig. 4). This result agrees with the information reported by Soto *et al*. (2017) in the frog *Agalychnis dacnicolor*, whose BT was significantly higher in grassland than in forest areas. This difference could be explained due to the low cover vegetation in open areas that may allow a major solar radiation and consequently upper temperatures (Pintanel *et al*. 2019; Scheffers *et al*. 2017), while the arboreal vegetation in forest habitats may act like a buffer that generates low temperatures (Pincebourde & Suppo 2016; Scheffers *et al*. 2017). Nevertheless, at the highest elevational zone studied (2,700 m.a.s.l), the difference of anuran BT between open and forest habitats was not statistically significant, even though the environmental temperature in open areas was slightly hotter than in forest habitats (Fig. 5). This similarity between open and forest environments at the highest elevation could be due to the fact that BTs of both studied species, *Niceforonia adenobrachia* and *P. permixtus*, were measured in equivalent microhabitat conditions (*N. adenobrachia*: under rocks, plants or soil vegetation; *P. permixtus*: on bushes or ground). As opposed to our hypothesis, the thermal variance of anuran BT from forest habitat was not statistically lower than that from open habitat for each elevational level studied (Fig. 4). We consider that this result can be attributed to the most heterogeneous landscape (and thus a slightly wider thermal variance) of the forest habitat where we recorded the anuran BT (on the ground, mosses, tree branches, tree leaves, in water bodies), in comparison to the open habitat that was most homogenous (leaf litter, rocks and the soil vegetation). It has been demonstrated that within a forest, the microclimates change according to features such as topography, landscape composition and local water balance (De Frenne *et al*. 2021).

Some species found at the same elevation in both forest and open habitats (*Pristimantis taeniatus* and *R. margaritifera*) had similar BTs (Table 1). Contrarily, *B. platanera* and *R. margaritifera* sampled along two elevational zones showed BTs more associated to their elevation than their habitat (Table 1). Consequently, variations in both microhabitat and elevational environmental temperatures are good predictors of BTs in anurans and then their knowledge is crucial to determine risks associated to climate changes and habitat loss, which are considered key threatening processes for the biodiversity, as they cause thermal stress and droughts (Mantyka *et al*. 2011). For instance, the rapid decline in a population of the salamander *Aneides aeneus* within a highly fragmented habitat has been linked with an increase in the environmental temperature (Corser 2001). This information is also particularly valuable in tropical habitats that are frequently exposed to rapid fragmentation and destruction (Alroy 2017), as many anuran species are adapted to the specific temperature of their microhabitats (Bernal & Lynch 2013), where narrow changes in these temperatures may seriously affect the survival of species (Turriago *et al*. 2015), some of them likely stenothermic (i.e., organisms that cannot tolerate wide ranges of environmental temperatures) (Navas *et al*. 2013).

## CONCLUSION

In this work, we found that:

1. Substrate temperature (Ts) is notably a better predictor of tropical anuran BT than a close air temperature (Ta) (approximately 20 cm above anurans). This information is important to be more accurate in predicting the risk of amphibians due to the global warming as many forecasts are obtained from macroenvironmental temperatures rather than those from microenvironmental habitats (Navas *et al*. 2013; Pintanel *et al*. 2019).
2. Anurans from high elevation (2,700 m.a.s.l), have a lower BT than those from lowlands (455 m.a.s.l), as expected by the temperature lapse rate. This result demonstrates the thermoconformity of anuran BT along elevational gradients in the Colombian Andes.
3. Anurans from open habitats have a greater mean, although not necessarily a wider BT variation, than the species from forest habitats. These data show that the temperature of habitats can change even within the same elevational level, microhabitats, and thus, the anuran BT.

This reinforces the importance of the records of microhabitat temperatures to a better estimation of anuran BTs, and the physiological and behavioural effects of habitat modification (e.g., deforestation, habitat conversion) on these ectotherms. Finally, given that anurans from forest habitat have a lower mean BT than species from open habitat, it would be interesting to test if anurans from these habitats are more thermally specialists with a lower thermal tolerance than those from open areas (see Pintanel *et al*. [2019] for an example with *Pristimantis* frogs). This may have conservation implications considering the substantial heating found in converted tropical forest and threatened environments including tropical Andes (Senior *et al*. 2017).

## Figures and Tables

**Table 1:** Body temperature (BT) of anurans, substrate (Ts) and air (Ta) temperatures for all sampled species in the open and forest habitats from three elevational zones in the Cordillera Central of Colombia (Tolima Department).

Elevational zone	Habitat	Species	*n*	BT (°C)	Ts (°C)	Ta (°C)
Mariquita: 455 m.a.s.l	Forest	*Pristimantis gaigei*	7	24.6 ± 1.20 (22.9–25.9)	25.0 ± 1.04 (23.6–26.5)	25.2 ± 1.07 (23.9–26.7)
Mariquita: 455 m.a.s.l	Forest	*Rhinella margaritifera*	13	23.9 ± 0.55 (22.5–24.8)	24.1 ± 0.54 (23.0–25.3)	24.6 ± 0.89 (23.2–26.0)
Mariquita: 455 m.a.s.l	Forest	*Boana platanera*	4	23.9 ± 0.59 (23.5–24.8)	24.1 ± 0.43 (23.8–24.7)	24.3 ± 0.47 (24.0–25.0)
		All anurans	24	24.1 ± 0.83 (22.5–25.9)	24.3 ± 0.80 (23.0–26.5)	24.7 ± 0.93 (23.2–26.7)
Mariquita: 455 m.a.s.l	Open	*Rhinella margaritifera*	14	25.2 ± 0.78 (23.7–26.8)	25.5 ± 0.64 (24.5–26.4)	25.9 ± 0.64 (24.4–26.8)
Mariquita: 455 m.a.s.l	Open	*Engystomops pustulosus*	6	25.0 ± 0.68 (24.0–26.0)	25.7 ± 0.60 (24.9–26.4)	24.2 ± 1.25 (22.4–25.8)
Mariquita: 455 m.a.s.l	Open	*Craugastor metriosistus*	3	25.0 ± 0.20 (24.8–25.2)	25.5 ± 1.05 (24.5–26.6)	26.1 ± 1.03 (25.0–27.0)
		All anurans	23	25.1 ± 0.69 (23.7–26.8)	25.5 ± 0.66 (24.5–26.6)	25.5 ± 1.14 (22.4–27.0)
Líbano: 1,400 m.a.s.l	Forest	*Pristimantis taeniatus*	13	19.1 ± 0.85 (18.0–20.3)	19.8 ± 1.08 (18.0–21.7)	19.8 ± 0.88 (18.3–21.4)
Líbano: 1,400 m.a.s.l	Forest	*Espadarana prosoblepon*	11	19.6 ± 0.85 (18.1–20.6)	20.9 ± 1.34 (19.2–22.6)	21.8 ± 1.36 (20.1–24.5)
Líbano: 1,400 m.a.s.l	Forest	*Rulyrana susatamai*	3	19.2 ± 1.19 (18.2–20.5)	20.1 ± 0.61 (19.7–20.8)	20.7 ± 0.23 (20.6–21.0)
		All anurans	27	19.3 ± 0.88 (18.0–20.6)	20.3 ± 1.24 (18.0–22.6)	20.7 ± 1.41 (18.3–24.5)
Líbano: 1,400 m.a.s.l	Open	*Boana platanera*	12	22.3 ± 0.42 (21.5–22.9)	22.5 ± 0.62 (21.4–23.6)	22.9 ± 0.80 (21.0–24.0)
Líbano: 1,400 m.a.s.l	Open	*Pristimantis taeniatus*	5	20.9 ± 1.12 (19.5–22.1)	21.4 ± 1.04 (20.1–22.6)	21.8 ± 0.90 (21.0–23.0)
Líbano: 1,400 m.a.s.l	Open	*Rhinella margaritifera*	3	22.4 ± 0.31 (22.1–22.7)	23.1 ± 0.67 (22.3–23.5)	23.6 ± 0.21 (23.4–23.8)
		All anurans	20	21.9 ± 0.90 (19.5–22.9)	22.3 ± 0.90 (20.1–23.6)	22.7 ± 0.97 (21.0 –24.0)
Murillo: 2,700 m.a.s.l	Forest	*Pristimantis boulengeri*	11	11.2 ± 0.88 (10.0–12.7)	11.2 ± 1.41 (8.9–12.9)	12.0 ± 1.58 (10.0–14.3)
Murillo: 2,700 m.a.s.l	Forest	*Niceforonia adenobrachia*	9	11.4 ± 0.99 (10.0–12.8)	11.3 ± 0.81 (10.2–12.5)	11.8 ± 0.73 (10.8–12.8)
Murillo: 2,700 m.a.s.l	Forest	*Pristimantis permixtus*	3	11.0 ± 0.87 (10.0–11.5)	11.3 ± 0.61 (10.7–11.9)	11.2 ± 0.95 (10.3–12.2)
		All anurans	23	11.2 ± 0.90 (10.0–12.8)	11.2 ± 1.09 (8.9–12.9)	11.8 ± 1.22 (10.0–14.3)
Murillo: 2,700 m.a.s.l	Open	*Pristimantis permixtus*	10	11.9 ± 1.09 (11.0–13.9)	12.3 ± 0.80 (11.8–14.3)	11.4 ± 1.15 (9.6–13.4)
Murillo: 2,700 m.a.s.l	Open	*Niceforonia adenobrachia*	8	11.8 ± 0.50 (11.0–12.5)	11.6 ± 0.91 (10.4–12.9)	11.6 ± 0.84 (10.5–13.3)
		All anurans	18	11.8 ± 0.86 (11.0–13.9)	12.0 ± 0.91 (10.4–14.3)	11.5 ± 1.00 (9.6–13.4)
		Total anurans	135			

*Notes*: Values represent mean, ± standard deviation, and range. °C = Celsius degree. *n* = number of individuals.

**Table 2:** Pearson correlations (r) of body temperature (BT) vs substrate (Ts) and air (Ta) temperatures for two habitats (open and forest) located at three elevational zones in the Cordillera Central of Colombia. *n*: number of individuals.

Elevational zone	Habitat	*n*	BT–Ts	BT–Ta
Mariquita: 455 m.a.s.l	Forest	24	r = 0.89; *p* < 0.000	r = 0.49; *p* < 0.016
Mariquita: 455 m.a.s.l	Open	23	r = 0.71; *p* < 0.000	r = 0.60; *p* > 0.789
Líbano: 1,400 m.a.s.l	Forest	27	r = 0.80; *p* < 0.000	r = 0.64; *p* < 0.000
Líbano: 1,400 m.a.s.l	Open	20	r = 0.65; *p* < 0.002	r = 0.54; *p* < 0.013
Murilo: 2,700 m.a.s.l	Forest	23	r = 0.76; *p* < 0.000	r = 0.61; *p* < 0.001
Murilo: 2,700 m.a.s.l	Open	18	r = 0.66; *p* < 0.003	r = 0.62; *p* < 0.006

## References

[b1-tlsr_35-1-219] Angilletta MJ (2009). Thermal adaptation a theoretical and empirical synthesis.

[b2-tlsr_35-1-219] Alroy J (2017). Effects of habitat disturbance on tropical forest biodiversity. Proceedings of the National Academy of Sciences.

[b3-tlsr_35-1-219] Angulo A, Rueda J, Rodriguez J, La Marca E (2006). Técnicas de inventario y monitoreo para los anfibios de la Región Tropical Andina.

[b4-tlsr_35-1-219] Armesto LO, Señaris JC (2017). Anuros del norte de los andes: Patrones de riqueza de especies y estado de conservación. Papéis Avulsos de Zoologia.

[b5-tlsr_35-1-219] Bernal MH, Lynch JD (2013). Thermal tolerance in anuran embryos with different reproductive modes: Relationship to altitude. The Scientific World Journal.

[b6-tlsr_35-1-219] Blomberg SP, Garland T, Ives AR (2003). Testing for phylogenetic signal in comparative data: Behavioral traits are more labile. Evolution.

[b7-tlsr_35-1-219] Bohlman S, Matelson T, Nadkarni N (1995). Moisture and temperature patterns of canopy humus and forest floor soil of a montane cloud forest, Costa Rica. Biotropica.

[b8-tlsr_35-1-219] Brattstrom BH (1963). A preliminary review of the thermal requirements of amphibians. Ecology.

[b9-tlsr_35-1-219] Carvajalino JM, Bonilla MA, Navas CA (2011). Freezing risk in tropical high-elevation anurans: An assessment based on the Andean frog *Pristimantis nervicus* (Strobomantidae). South American Journal of Herpetology.

[b10-tlsr_35-1-219] Catenazzi A, Lehr E, Vredenburg VT (2014). Thermal physiology, disease, and amphibian declines on the Eastern slopes of the Andes. Conservation Biology.

[b11-tlsr_35-1-219] Corn PS (2005). Climate change and amphibians. Animal Biodiversity and Conservation.

[b12-tlsr_35-1-219] Corser JD (2001). Decline of disjunct green salamander (*Aneides aeneus*) populations in the southern Appalachians. Biological Conservation.

[b13-tlsr_35-1-219] Cruz EX, Galindo CA, Bernal MH (2016). Dependencia térmica de la salamandra endémica de Colombia *Bolitoglossa ramosi* (Caudata, plethodontidae). Iheringia – Série Zoologia.

[b14-tlsr_35-1-219] De Frenne P, Lenoir J, Luoto M, Scheffers B, Zellweger F, Aalto J, Ashcroft M, Christiansen D, Decocq G, Pauw K, Govaert S, Greiser C, Gril E, Hampe A, Tommaso J (2021). Forest microclimates and climate change: Importance, drivers and future research agenda. Global Change Biology.

[b15-tlsr_35-1-219] Delgado P, Burrowes PA (2022). Response to thermal and hydric regimes point to differential inter- and intraspecific vulnerability of tropical amphibians to climate warming. Journal of Thermal Biology.

[b16-tlsr_35-1-219] Duellman WE, Trueb L (1994). Biology of amphibians.

[b17-tlsr_35-1-219] Ehlers TA, Poulsen CJ (2009). Influence of Andean uplift on climate and paleoaltimetry estimates. Earth and Planetary Science Letters.

[b18-tlsr_35-1-219] FAO (2001). Causas y tendencias de la deforestación en América Latina.

[b19-tlsr_35-1-219] Feder ME, Lynch JF (1982). Effects of latitude, season, elevation, and microhabitat on field body temperatures of neotropical and temperate zone salamanders. Ecology.

[b20-tlsr_35-1-219] Galeana JM, Corona N, Ordóñez JA (2009). Análisis dimensional de la cobertura vegetal-uso de suelo en la cuenca del río Magdalena. Ciencia Forestal En México.

[b21-tlsr_35-1-219] Gates DM (1980). Biophysical ecology.

[b22-tlsr_35-1-219] Hillman SS, Withers PC, Drewes RC, Hillyard SD (2008). Ecological and environmental physiology of amphibians.

[b23-tlsr_35-1-219] Hussain QA, Pandit AK (2012). Global amphibian declines: A review. International Journal of Biodiversity and Conservation.

[b24-tlsr_35-1-219] Iturra M, Vidal M, Labra A, Ortiz JC (2014). Winter thermal ecology of *Pleurodema thaul* (Amphibia: Leptodactylidae). Gayana.

[b25-tlsr_35-1-219] Jones MM, Szyska B, Kessler M (2011). Microhabitat partitioning promotes plant diversity in a tropical montane forest. Global Ecology and Biogeography.

[b26-tlsr_35-1-219] Khatiwada JR, Zhao T, Jiang J (2020). Variation of body temperature of active amphibians along elevation gradients in eastern Nepal Himalaya. Journal of Thermal Biology.

[b27-tlsr_35-1-219] Lara RA, Luja VH (2018). Body temperatures of some amphibians from Nayarit, Mexico. Revista Mexicana de Biodiversidad.

[b28-tlsr_35-1-219] Leyte A, Gonzáles RL, Quintero GE, Alejo F, Berriozabal C (2018). Aspectos ecológicos de una comunidad de anuros en un ambiente tropical estacional en Guanajuato, México. Acta Zoológica Mexicana.

[b29-tlsr_35-1-219] Lynch JD, Suárez A (2002). Análisis biogeográfico de los anfibios paramunos. Caldasia.

[b30-tlsr_35-1-219] Mantyka C, Martin T, Rhodes J (2011). Interactions between climate and habitat loss effects on biodiversity: A systematic review and meta-analysis. Global Change Biology.

[b31-tlsr_35-1-219] Navas CA, Carvajalino JM, Saboyá LP, Rueda LA, Carvajalino MA (2013). The body temperature of active amphibians along a tropical elevation gradient: patterns of mean and variance and inference from environmental data. Functional Ecology.

[b32-tlsr_35-1-219] Nowakowski AJ, Watling JI, Thompson ME, Brusch GA, Catenazzi A, Whitfield SM, Kruz DJ, Suarez A, Aponte A, Donnelly MA, Todd BD (2018). Thermal biology mediates responses of amphibians and reptiles to habitat modification. Ecology Letters.

[b33-tlsr_35-1-219] Nowakowski AJ, Watling JI, Whitfield SM, Todd BD, Kurz DJ, Donnelly MA (2017). Tropical amphibians in shifting thermal landscapes under land-use and climate change. Conservation Biology.

[b34-tlsr_35-1-219] Oromí N, Sanuy D, Sinsch U (2010). Thermal ecology of natterjack toads (*Bufo calamita*) in a semiarid landscape. Journal of Thermal Biology.

[b35-tlsr_35-1-219] Ortiz CE, Páez V, Zapata FA (2013). Temperature and precipitation as predictors of species richness in northern Andean amphibians from Colombia. Caldasia.

[b36-tlsr_35-1-219] Pincebourde S, Suppo C (2016). The vulnerability of tropical ectotherms to warming is modulated by the microclimatic heterogeneity. Integrative and Comparative Biology.

[b37-tlsr_35-1-219] Pintanel P, Tejedo M, Ron SR, Llorente GA, Merino A (2019). Elevational and microclimatic drivers of thermal tolerance in Andean *Pristimantis* frogs. Journal of Biogeography.

[b38-tlsr_35-1-219] Pough FH, Andrews RM, Crump ML, Savitzky AH, Wells KD, Brandley MC (2015). Herpetology.

[b39-tlsr_35-1-219] Ramírez S, Meza P, Yánez M, Reyes J (2009). Asociaciones interespecíficas de anuros en cuatro gradientes altitudinales de la Reserva Biológica Tapichalaca, Zamora-Chinchipe, Ecuador. Serie Zoológica.

[b40-tlsr_35-1-219] Root TL, Price JT, Hall KR, Schneider SH, Rosenzweig C, Pounds JA (2003). Fingerprints of global warming on wild animals and plants. Nature.

[b41-tlsr_35-1-219] Rueda LA, Navas CA, Carvajalino JM, Amézquita A (2016). Thermal ecology of montane *Atelopus* (Anura: Bufonidae): A study of intrageneric diversity. Journal of Thermal Biology.

[b42-tlsr_35-1-219] Sanabria EA, Quiroga LB, Acosta JC (2003). Ecología térmica de *Leptodactylus ocellatus* (Linnaeus, 1758) (Anura: Leptodactylidae) en los bañados de zonda, San Juan, Argentina. Cuadernos de Herpetología.

[b43-tlsr_35-1-219] Sanabria EA, Quiroga LB, Martino AL (2011). Seasonal changes in the thermoregulatory strategies of *Rhinella arenarum* in the Monte desert, Argentina. Journal of Thermal Biology.

[b44-tlsr_35-1-219] Sanabria EA, Vaira M, Quiroga LB, Akmentins MS, Pereyra LC (2014). Variation of thermal parameters in two different color morphs of a diurnal poison toad, *Melanophryniscus rubriventris* (Anura: Bufonidae). Journal of Thermal Biology.

[b45-tlsr_35-1-219] Sanabria E, Quiroga L (2019). The body temperature of active desert anurans from hyper-arid environment of South America: The reliability of WorldClim for predicted body temperatures in anurans. Journal of Thermal Biology.

[b46-tlsr_35-1-219] Scheffers BR, Edwards DP, Macdonald SL, Senior RA, Andriamahohatra LR, Roslan N, Rogers A, Haugaasen T, Wright P, Williams SE (2017). Extreme thermal heterogeneity in structurally complex tropical rain forests. Biotropica.

[b47-tlsr_35-1-219] Senior RA, Hill JK, González P, Goode LK, Edwars DP (2017). A pantropical analysis of the impacts of forest degradation and conversion on local temperature. Ecology and Evolution.

[b48-tlsr_35-1-219] Soto Y, Suazo I, Urbina N, Marroquín J, Alvarado J (2017). Efecto de los estadios sucesionales del bosque tropical seco sobre el microhábitat usado por *Agalychnis dacnicolor* (Anura: Phyllomedusidae) y *Smilisca fodiens* (Anura: Hylidae). Revista de Biología Tropical.

[b49-tlsr_35-1-219] Taylor E, Diele L, Gangloff E, Hall J, Halpern B, Massey M, Rödder D, Rollinson N, Spears S, Sun B, Telemeco R (2020). The thermal ecology and physiology of reptiles and amphibians: A user’s guide. Journal of Experimental Zoology.

[b50-tlsr_35-1-219] Turriago J, Parra C, Bernal M (2015). Upper thermal tolerance in anuran embryos and tadpoles at constant and variable peak temperatures. Canadian Journal of Zoology.

